# Gaussian mixture model for enhancing the quality of transmission estimation in optical networks: a machine learning approach

**DOI:** 10.1038/s41598-025-27355-5

**Published:** 2025-12-09

**Authors:** Shakrajit Sahu, J. Christopher Clement

**Affiliations:** School of Electronics Engineering, Vellore Institute of Technology, Vellore, Tamil Nadu 632014 India

**Keywords:** Fiber-optic communications, Machine learning, Gaussian mixture model, Bit error rate, Signal-to-noise ratio, Engineering, Mathematics and computing

## Abstract

In recent times, there has been a huge amount of work on utilizing deep learning (DL) to estimate the quality of transmission (QoT) in optical networks. This research depict a lightpath’s quality of transmission to develop advanced fiber-optic communication and networks based on DL technique. We need different primary estimation parameters for advanced optical fiber communication and networks, i.e., modulation formats, baud rate, and code rate.Recently, the QoT for unspecified optical paths relies on various estimation approaches i.e., (1) analytical models estimating physical layer impairments (PLIs) and (2) margined formulas. This paper emphasis on Gaussian mixture model (GMM) based algorithm that can be applied to optimization and sophisticated systems. The model can forecast bit-error rate, and signal-to-noise ratio (SNR) of unknown optical paths with threshold value, traffic volume, and modulation format. The model was trained and tested using features from Korean network topology. The Area Under the ROC Curve (AUC) from the simulated outcome is 1.00, while maintaining the high accuracy, F1 score, low Brier score and low expected calibration error (ECE).

## Introduction

Advances in optical communications using coherent technology have revolutionized the industry, offering various design possibilities for lightpath deployment ^1^. Modulation formats, adaptable channel spacing, and flex-grid technologies provide the system and network engineers with degrees of freedom and produce numerous combinations of lightpaths. Lightpath quality of transmission (QoT) predictability prior to deployment is absolutely necessary for optimum planning of the optical networks. Present estimation mechanisms belong to the following two paradigms. Physical layer limitations are accurately expressed in analytical models such as Split-Step Fourier, but since they need copious amounts of computation, even real-time extrapolations in real-sized networks could be difficult to perform. Low-computation intensive approximated functions, on the other hand, provide greater margin in links and thereby have less error tolerance with resulting suboptimality in resources in the optical networks ^2^. This approach examines machine learning methods like network kriging, case-based reasoning, and neural networks to learn from experience. Another approach uses the quantification of QoT of lightpaths by optical performance monitors (OPMs) to forecast the QoT of non-established paths ^3^, ^4^. Forecast the probability that the Bit Error Rate (BER) of a lightpath is below the system threshold based on traffic volume, modulation scheme, lightpath distance, longest link, and number of links ^5^. The classifier is trained using actual BER measurements from optical performance monitors or simulated data from an E-Tool BER estimation tool because field data is hard to obtain ^6^. In the final deployment decision, the classification output is intended to support a routing and spectrum assignment (RSA) method ^7^. Emphasizes performance measurement and effect on classification accuracy with respect to training data. Suggests the requirement of light path probes in order to measure non-standard user configurations of field BER to ensure verification of optimum performance since existing measurements might not provide optimum results ^8^. Fesl et al. proposed a Gaussian Mixture Model (GMM)-based channel estimator that learned from imperfect training data, i.e., noisy and sparsely distributed pilot observations. The expectation-maximization algorithm they designed with modifications offered improved robustness against training data imperfections with performance closely following that obtained using perfect channel state information ^9^. Another piece of work by Fesl et al. involved channel estimation with structured covariance matrices, where they proposed GMM-based estimators that achieved performance versus computational efficiency trade-offs. Their work helped extend estimation accuracy with practical feasibility in large-scale networks ^10^. Baud rate is the frequency of changes of the signal or symbols per second in a communication optical channel. The bit rate refers to bits per second being transmitted, which predicts the amount of data in the future. The bit rate is greater than the baud rate since more than one bit may be represented by a symbol.

Turan et al. applied GMMs to describe the probability density function of mobile terminal channel trajectories. Their method allowed for successful channel prediction without Signal-to-Noise Ratio (SNR)-specific training, and it outperformed current approaches ^11^. Weiayer et al. designed techniques using data symbols together with pilot symbols to improve channel estimation for multi-user systems. With the help of GMM-based estimators, they attained better estimation quality with lower computational complexity ^12^. Tian et al. proposed a GMM-Hidden Markov Model-based equalizer to counteract nonlinear distortions in optical fiber networks. Their scheme greatly minimized the BER while decreasing the computational complexity by about 73% in comparison with recurrent neural network-based solutions ^13^.

Optical communication networks are the backbone of contemporary telecommunications, facilitating data transmission over high speed and long distances with low latency. With growing demands for high-bandwidth applications like 5G networks, cloud computing, and streaming services in real time, achieving optimal performance in optical networks is a top priority challenge. Two intrinsic parameters to assess network performance are BER and SNR, which have direct implications on data integrity, quality of transmission, and network efficiency overall. BER measures the likelihood of bit errors in data transmission, whereas SNR estimates the signal power to noise power ratio, indicating the quality of received signals. Both of these parameters are estimated by several physical and operational parameters such as traffic load, transmission rate, modulation methods, and network structure. Proper estimation of BER and SNR is essential for optimizing network settings, reducing errors, and providing high Quality of Service (QoS).

Traditional methods for BER and SNR estimation tend to be based on analytical models and empirical measurements, which can be computationally demanding and inflexible. Machine learning-based models offer a more adaptive and data-driven solution, enabling predictions based on observed trends in the data instead of pre-defined equations. Among the models, the GMM provides a strong probabilistic model for estimating BER and SNR by modeling complex data distributions as a mixture of multiple Gaussian components. The method models non-linear interactions between input features and network performance measures effectively. In this paper, we propose a GMM-based methodology to estimate BER and SNR using five key input features: traffic volume, number of links, modulation constellation, transmission speed, and link length. Our model categorizes network conditions into multiple Gaussian components, enabling accurate performance estimation for unknown network paths. By leveraging probabilistic modeling, the proposed approach enhances decision-making processes in optical communication networks, offering a scalable and adaptable solution for real-world deployment.

The rest of our paper is structured as follows: In Section 2 reviews related studies. Section 3 details the proposed methodology, including the problem formulation and GMM framework. Section 4 presents experimental results, evaluating the effectiveness of our approach against traditional methods. Section 5 discusses findings and their implications for optical network optimization. Lastly, Section 6 concludes the paper and provides directions for the future.

## Literature survey

GMMs were in the limelight a few years ago with their capacity to represent complex distributions and thus were found to be highly appropriate for many applications in optical communication systems and relevant disciplines. One of the main areas where GMMs have been used is the study of nonlinear phenomena in optical networks. Sedov et al. explored how GMMs can accurately represent nonlinear effects in optical communication systems, proving that multi-component Gaussian distributions provide an accurate description of signal fluctuations. Their research confirmed that GMMs improve signal behavior modeling accuracy, especially in situations with nonlinear impairments, enhancing network reliability and performance ^14^. Building on the use of GMMs in optical networks, Zhao et al. proposed a hybrid method by combining GMMs with Hidden Markov Models to design a nonlinear equalizer for optical fiber transmission. Their approach efficiently suppressed fiber nonlinear distortions and achieved great enhancements in signal quality and overall transmission performance. This work highlighted the strength of GMM-based models in addressing nonlinear distortions and placed them at the forefront of future high-speed optical communication systems ^15^. Outside of optical communications, GMMs have also been successful in electrical network diagnosis. Mas’ud et al. utilized GMMs for classifying various stages of growth of an electrical tree in epoxy resin insulation. Their work illustrated that GMM-based classification enhanced the accuracy of detecting insulation degradation stages, an important consideration in predictive maintenance and fault management of electrical networks. Through probabilistic modeling, the method facilitated enhanced asset management techniques, mitigating the possibility of sudden failure ^16^. Besides their use in optical and electrical systems, GMMs have also been utilized in feature fusion in object detection tasks. Li et al. came up with a better GMM method for object moving object segmentation, especially for dynamic scenes with visual distortions. Their system efficiently improved the clarity of contours while reducing noise, resulting in increased detection precision in real-time applications. The research pointed to the versatility of GMMs across different applications, further underscoring their applicability in data-driven modeling applications ^17^. For laser amplitude and phase noise estimation, Bayesian filtering is used. A k-nearest neighbors (KNN) approach reduces impairments such as non-Gaussian symmetric noise, laser phase noise, and nonlinear phase noise in zero-dispersion and dispersion-managed fibers. For transmission quality estimation between lightpaths in terms of BER, we use the best regression algorithms: network kriging and least-squares minimization with $L_2$-norm regularization ^18^. The BER is estimated at lightpath nodes by optical performance monitors. Multiple routes and modulation schemes are utilized for set lightpaths. A deep learning classifier determines the highest probability with a BER value less than the threshold value. For the estimation of nonlinear effects, the optimal BER calculation model is identified with different training datasets. Dynamic routing and spectrum assignment with first-fit criterion algorithms are used for different network topologies and classification features ^19^.

Collectively, these studies demonstrate the wide-ranging potential of GMMs to enhance system performance across optical communication, nonlinear equalization, predictive maintenance, and object detection. Their capacity to handle intricate statistical relations makes them a valuable asset in solving fundamental issues in contemporary technological systems. The findings from these studies form a sound basis for further development in BER and SNR estimation for optical networks, guaranteeing greater efficiency and dependability in communications systems.

## BER estimation and nonlinearity management in optical networks

Prior to describing our BER estimation tool (E-Tool), we must establish its system model for generating precise estimates. Our grid employs 12.5 GHz slices with agile transceivers at 28 Gbaud in a 37.5 GHz bandwidth (3 slices). Super-channels group transceivers for higher traffic. We employ single-mode fibers with no dispersion compensation, amplified signal power from 100 km spaced optical amplifiers (20 dB gain, 5 dB noise figure). Coherent detection, analog-to-digital conversion, electronic chromatic dispersion compensation, and an adaptive equalizer are employed in each receiver for linear impairments. We count errors to calculate the pre-FEC BER. The E-Tool accepts network topology, traffic demands, and modulation formats as inputs. Its process utilizes deep learning for data input to QoT estimation ^20^, ^21^. It provides outputs like BER and signal-to-noise ratio as predictors of transmission quality by deep learning. The tool is dynamic and static network adaptive, and it accommodates various topologies and traffic patterns. It is trained on real optical conditions such as noise and nonlinear effects to make accurate QoT predictions. Studies validate it is superior to traditional analytical models in complex networks.

BER estimation is critical to the performance of an optical network. The E-Tool provides accurate pre-Forward Error Correction (pre-FEC) BER estimation as a function of lightpath characteristics and modulation format ?. Forward Error Correction raises the pre-FEC BER to an acceptable value if the pre-FEC BER is lower than a certain threshold. Pre-FEC BER is related to SNR in systems with chromatic dispersion and AWGN, and target BER is used to estimate the required SNR for error-free lightpath. A link budget calculates transmitted power, gains, and losses to ensure the signal reaches the target SNR for error-free transmission ^22^.

Optical fiber transmission is plagued by nonlinear perturbations because of channel interactions. Power-Nonlinear-Interference (PNLI) is added as a white Gaussian noise component to mitigate these effects. This makes quantification and minimization of nonlinear distortions possible, along with accurate estimation of BER and enhanced network performance. The incorporation of BER prediction, SNR estimation, and PNLI modeling enables optical networks to achieve optimal signal integrity and transmission efficiency.

## Problem statement

The goal of this research work is to create a data-driven and effective method to estimate BER and SNR in optical communication networks. As these parameters are random, multimodal, and heterogeneous in nature, the conventional methods of estimation are not effective regarding generalizability. For the purpose of surpassing this, a method with the help of GMM is introduced, where a probabilistic model is utilized to study the complex relationship between the important parameters of the network and the transmission quality.

The method addresses five major input parameters that significantly influence BER and SNR:

- Traffic Volume (amount of traffic transported along a lightpath).
- Number of Links (total number of links traversed by the lightpath).
- Modulation Constellation (such as QPSK, 16-QAM).
- Transmission Speed (data rate in Gbps).
- Link length (physical distance along a link in km).

By estimating such parameters using GMM, the proposed method properly classifies network conditions into various performance classes (Low-Grade Performance, High-Speed Transmission, and High-Quality Service). This enables more accurate prediction of BER and SNR, improved network monitoring, and improved optical network performance optimization.

## Proposed method

In optical communication systems, the accurate estimation of BER and SNR is required to evaluate network performance. This methodology employs a GMM for estimating BER ($y_1$) and SNR ($\gamma$) from five input features into two output features, while the input features are in regard to physical and operational characteristics of the optical network, and the output features are the BER and SNR. The GMM is used to model the relationship between the input features and the output metrics, which enables prediction for an unspecified path.

### Problem formulation

The problem involves estimating two key performance metrics, the bit error rate ($y_1$) and the signal-to-noise ratio ($\gamma$), using four input features: traffic volume ($f_1$), which represents the total data transferred over the network; the modulation constellation ($f_2$), which refers to the type of modulation technique employed; transmission speed ($f_3$), representing the rate at which data is transmitted; and the length of a link ($f_4$), which denotes the physical distance of a link. The output features include the bit error rate ($y_1$), ranging from $10^0$ to $10^{-1}$, and the signal-to-noise ratio ($\gamma$), ranging from $-5$ dB to $-10$ dB. It comprises seven features in total, namely five input features and two output features. All the features are assumed to be independent of each other.

### Gaussian mixture model (GMM)

A GMM is a probabilistic model based on the assumption that the data are generated through a mixture of K Gaussian distributions. There are Gaussian components which correspond to various network conditions, categorized as: (1) Low-Grade Performance where Poor network condition in terms of very high BER and low SNR. (2) In High-Speed Transmission data transmission rates are high with an average BER and SNR. (3) High-Quality Service where Optimized network conditions, low BER and high SNR.

The joint probability density function for the input features $F = [f_1, f_2, f_3, f_4]$ and output features $Y = [y_1, \gamma ]$ is given by:

1 $$\begin{aligned} P(F, Y) = \sum _{k=1}^K \alpha _k \cdot \mathcal {N}([F, Y] | \textbf{m}_k, \textbf{C}_k) \end{aligned}$$

(1) expresses the probability of observing input features *F* and output features *Y* as a weighted sum of K Gaussian distributions, where each component has its mean vector $\textbf{m}_k$ and covariance matrix $\textbf{C}_k$, and the mixing coefficient $\alpha _k$. Also, in (1), $\mathcal {N}([F, Y] | \textbf{m}_k, \textbf{C}_k)$ indicates the joint density function with mean vector $\textbf{m}_k$ and covariance matrix $\textbf{C}_k$.

### Input feature modeling

Each input feature is represented by a Gaussian density function, allowing the model to capture the statistical behavior of the network parameters. Specifically, let $f_1$ represent the traffic volume, modeled as a Gaussian distribution with mean $\mu _1$ and variance $\sigma _{f1}^2$. The modulation constellation, denoted by $f_2$, follows a Gaussian distribution with mean $\mu _2$ and variance $\sigma _{f2}^2$. For transmission speed, $f_3$ captures its distribution with parameters $\mu _3$ and $\sigma _{f3}^2$. Finally, $f_4$ models the link length, governed by a Gaussian distribution with mean $\mu _4$ and variance $\sigma _{f4}^2$. This helps the Gaussian density functions capture statistical properties of input features, therefore enabling the GMM to model the relationships to the output features.

The mean vector $\textbf{m}_k$ for the *k*-th Gaussian component is given by $\textbf{m}_k = \left[ \mu _{f1}^{(k)} , \mu _{f2}^{(k)}, \mu _{f3}^{(k)} , \mu _{f4}^{(k)} , \mu _{y1}^{(k)} , \mu _{\gamma }^{(k)} \right] ^T$ which is the *k*-th Gaussian component and is the concatenation of the mean values of input features ($\mu _{f1}^{(k)}, \mu _{f2}^{(k)}, \cdots , \mu _{f4}^{(k)}$) and output features ($\mu _{y1}^{(k)}, \mu _{\gamma }^{(k)}$) .

The covariance matrix $\textbf{C}_k$ for the *k*-th Gaussian component is defined as the diagonal matrix $\textbf{C}_k = {\textbf {diag}} \left( \sigma _{f1}^2, \sigma _{f2}^2, \sigma _{f3}^2, \sigma _{f4}^2, \sigma _{y1}^2 ,\sigma _{\gamma }^2 \right)$, whose diagonal elements correspond to the variance of the feature ($\sigma _{f1}^2, \sigma _{f2}^2, \dots , \sigma _{\gamma }^2$) of the *k*-th Gaussian component. The off-diagonal elements are zero, implying that the features are independent.

### Conditional probability for prediction

To predict the output features $Y = [y_1, \gamma ]$ given the input features *F*, we compute the conditional probability:

2 $$\begin{aligned} P(Y | F) = \frac{P(F, Y)}{P(F)} \end{aligned}$$

while (2) is used to compute the conditional probability in regard to output features *Y* given the input features *F*, it is derived from the joint probability *P*(*F*, *Y*) and the marginal probability *P*(*F*).

### Implementation steps

There are three steps, namely training ,updation and Prediction involved in the implementation. They are discussed in detail below.

#### Step 1: data collection

The dataset consists of two main components: input features and output features. The input features are denoted as ($F = [f_1, f_2, f_3, f_4]$), which include traffic volume, modulation constellation, transmission speed, and link length. The output features are represented as ($Y = [y_1, \gamma ]$), corresponding to the bit error rate (BER) and signal-to-noise ratio (SNR).

#### Step 2: train the GMM

The GMM is trained to model the joint distribution *P*(*F*, *Y*) using $K = 3$ Gaussian components. The training process involves the Expectation-Maximization (EM) algorithm, which iteratively estimates the parameters of the GMM. There are two steps, namely E-step and M-step involved in the training process.

*E-Step: Responsibility Computation

For each data point $(F_i, Y_i)$, compute the responsibility $\beta _{i,k}$, which represents the probability that the data point belongs to the *k*-th Gaussian component:

3 $$\begin{aligned} \beta _{i,k} = \frac{\alpha _k \cdot \mathcal {N}([F_i, Y_i] | \textbf{m}_k, \textbf{C}_k)}{\sum \limits _{j=1}^K \alpha _j \cdot \mathcal {N}([F_i, Y_i] | \textbf{m}_j, \textbf{C}_j)} \end{aligned}$$

This step computes the probability of every data point for each Gaussian.

***M-Step: Parameter Update***

In this step, the parameters $\alpha _k$, $\textbf{m}_k$, and $\textbf{C}_k$ for each Gaussian component are updated as follows:

4 $$\begin{aligned} \alpha _k = \frac{\sum \limits _{i=1}^N \beta _{i,k}}{N} \end{aligned}$$

The mixing coefficient $\alpha _k$ is recalculated as the mean responsibility assigned to the $k$-th component.

5 $$\begin{aligned} \textbf{m}_k = \frac{\sum \limits _{i=1}^N \beta _{i,k} [F_i, Y_i]}{\sum \limits _{i=1}^N \beta _{i,k}} \end{aligned}$$

The mean vector $\textbf{m}_k$ is determined by computing the responsibility-weighted average of the data points.

6 $$\begin{aligned} \textbf{C}_k = \frac{\sum \limits _{i=1}^N \beta _{i,k} ([F_i, Y_i] - \textbf{m}_k)([F_i, Y_i] - \textbf{m}_k)^T}{\sum \limits _{i=1}^N \beta {i,k}} \end{aligned}$$

The covariance matrix $\textbf{C}_k$ is updated using the weighted covariance of the data points based on the corresponding responsibilities.

#### Step 3: prediction

For a new input $F_{\text {new}} = [f_1, f_2, f_3, f_4]$, the output $Y_{\text {new}} = [y_1, \gamma ]$ is predicted using the conditional probability:

7 $$\begin{aligned} P(Y | F_{\text {new}}) = \sum _{k=1}^K \beta _{k} \cdot \mathcal {N}(Y | \textbf{m}_{Y|k}, \textbf{C}_{Y|k}) \end{aligned}$$

Equation (7) predicts the output features *Y* for a new input $F_{\text {new}}$ by combining the contributions of all *K* Gaussian components.

### Summary of methodology

This method uses a GMM to approximate the BER and SNR in optical communication networks. The GMM is trained on a data set with five input features and two output features, with $K = 3$ Gaussian components that capture different network conditions. The EM algorithm is applied to iteratively estimate the model parameters so that accurate predictions of BER ($y_1$) and SNR ($\gamma$) can be made for new input data. This approach, therefore, gives a robust method for analyzing and optimizing the performance of optical networks in varying conditions with the following key contributions.

- *Independent features*: It is further assumed that all input and output features are independent, thereby reducing the modeling task.
- *GMM for network conditions*: The model can capture the three Gaussian components, which allows it to represent different network conditions, such as low-grade performance, high-speed transmission, and high-quality service.
- *Conditional probability for prediction*: The conditional probability framework ensures proper estimation of BER and SNR for new input data.

The method can be easily extended to other communication systems where the performances have to be estimated on the basis of operational and physical characteristics.

**Fig. 1:**
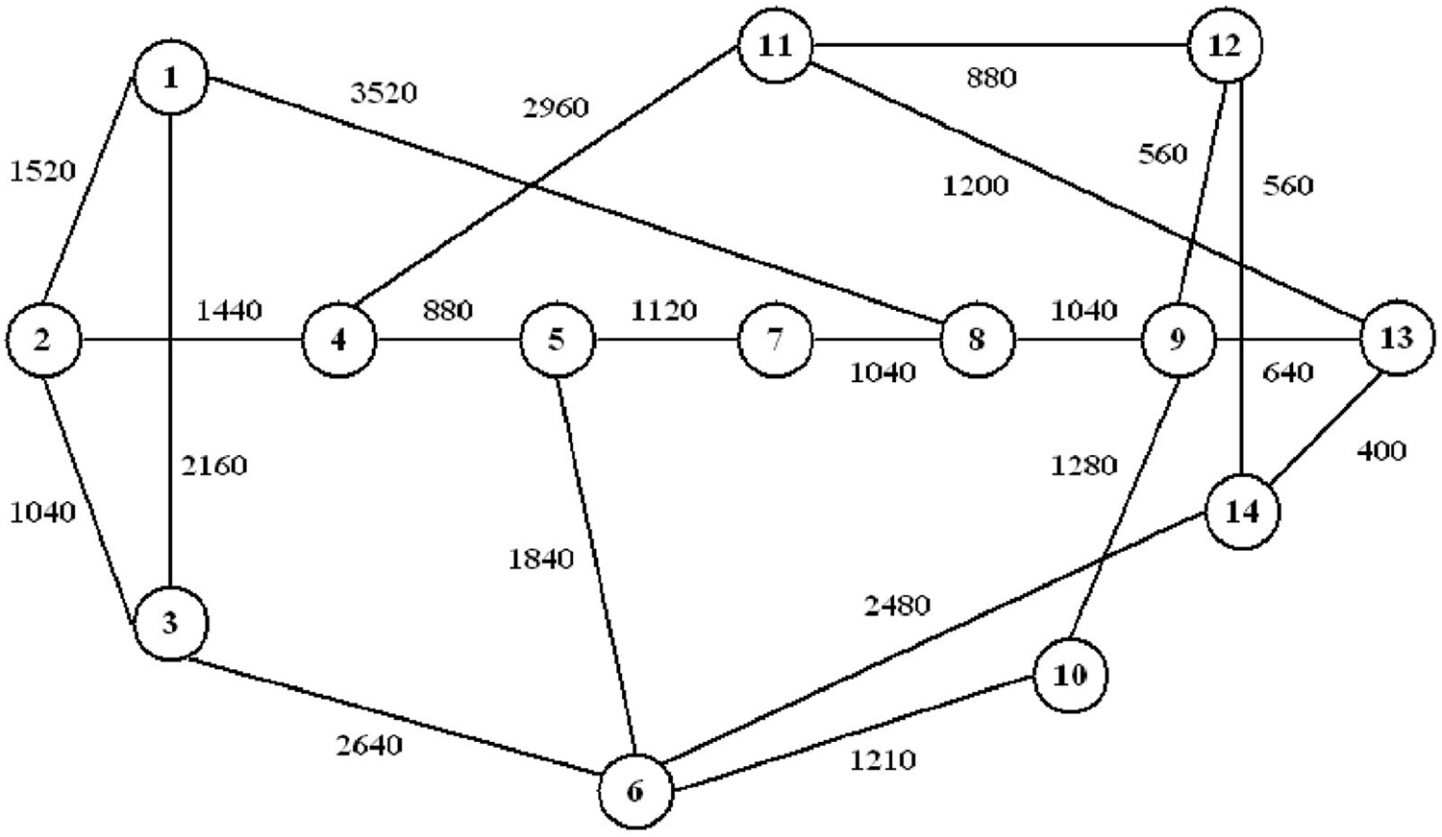
Korean network topology representation with 21 bidirectional links and 14 nodes.

**Fig. 2:**
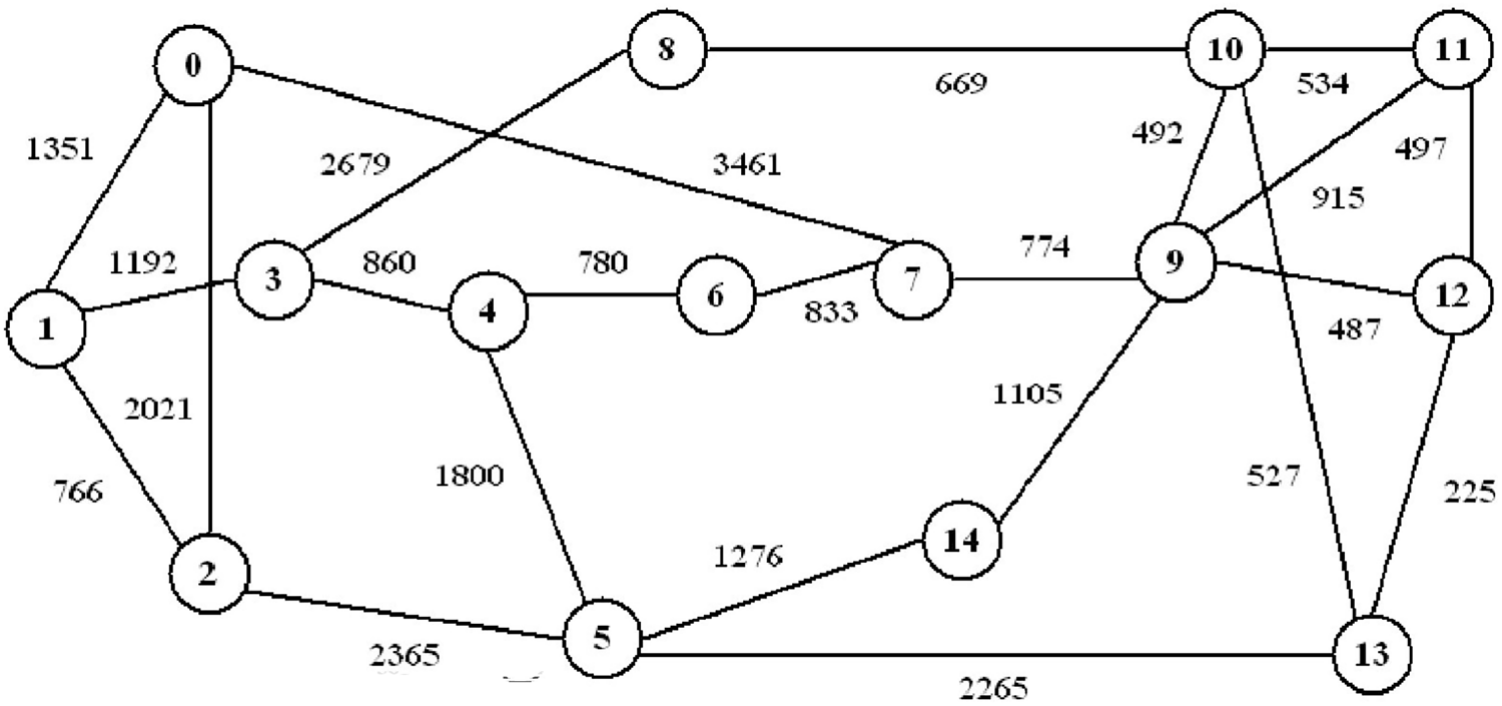
Representation of Korean network topology with 23 bidirectional links and 15 nodes.

### Dataset creation

The dataset creation adopts the synthetic data for effectual analysis. The network which was considered for the analysis is shown in Fig. 1 and 2. Firstly, the network makes lightpath routing and spectrum allocation by adopting margined computation then it uses only lightpath, which is authentic BER deployment for the training process. The dataset comprises of various features i.e., signal power, noise levels, and modulation formats. The independence between variables cannot be resolved by their units of measurement alone. Independence indicates the lack of a statistical relationship between variables, the value of one variable does not contribute information about the other. While units of measurement speculate the scale of the data, they do not have implicit independence. We need to use statistical methods, i.e., correlation analysis, to get the measure of whether variables are independent.

### Dataset generation

The accuracy and quality of the GMM used in $y_1$ and $\gamma$ estimation would be based on the training data. Real-world data for different network topologies may be hard to come by, particularly for heterogeneous optical network environments. Synthetic data will thus be created using python simulations, which will simulate signal propagation in an optical network. The dataset will contain four input parameters: traffic volume, modulation scheme, transmission speed, and link length. Python will be used to simulate various network conditions and calculate the respective $y_1$ and $\gamma$ values, taking into account linear and nonlinear distortions, and Gaussian noise. The dataset generated will comprise a very large range of network conditions, from normal conditions to extreme conditions, so the GMM would be trained on a large range of conditions. In this method, it is feasible to make an accurate prediction of $y_1$ and $\gamma$ even for new, never-seen network settings.

## Results and discussion

The simulation is performed using python as it includes libraries for different machine learning models. The dataset is generated and is used for training the GMM and other models as highlighted in the previous sections. The models are trained on a dataset with four input features, namely, traffic volume, modulation scheme, transmission speed, link length and two output features, namely, SNR and BER. The core of the simulation lies in the running of loops which runs over 100 trial, each time with a different seed. This will ensure that the results are not due to the influence of single lucky data set but involve different sets. Using a stratified split, the data is spilt into a training and test data, which maintains the proportion of good and failed links in both sets. In fact, 70 % of data is split as training set and 30 % of data is split as test set. At the begining, hypothetically, we assume that the data is independent and its correlation matrix is a diagonal one.

**Table 1:** Feature correlation matrix.

**Feature**	$f_1$	$f_2$	$f_3$	$f_4$	$y_1$	$\gamma$
$f_1$	1.0000	-0.0221	-0.0042	0.0348	0.5812	-0.6146
$f_2$	-0.0221	1.0000	-0.4709	-0.0058	-0.0127	0.0223
$f_3$	-0.0042	-0.4709	1.0000	0.0065	-0.0027	-0.0096
$f_4$	0.0348	-0.0058	0.0065	1.0000	0.7059	-0.6597
$y_1$	0.5812	-0.0127	-0.0027	0.7059	1.0000	-0.9387
$\gamma$	-0.6146	0.0223	-0.0096	-0.6597	-0.9387	1.0000

In order to verify the data independency, the correlation analysis of the data is performed. The correlation analysis of the data set is shown in Table 1, which shows that the independency assumption is weak. The pairs ‘link length’ and ‘BER’ exhibit a strong positive correlation, which indicates that longer link lengths might increase a BER. In addition, there is a moderate positive correlation between traffic volume and BER. This indicates that high traffic volume can increase an overall network load and, thereby, increases BER. There is a moderate negative correlation between the modulation scheme and the transmission speed. As the discrete modulation scheme index increases, from QPSK to 16-QAM and then to 64-QAM, the required analog transmission speed increases. However, since the scheme was encoded as 0,1 and 2, and the speed increases, the relationship appears negative depending on the specific encoding. Similarly, there is a strong negative correlation between SNR and BER, which is very obvious, as they are inversely related. As the off-diagonal cells of the matrix in Table 1 are non-zero, the assumption of the diagonal covariance matrix will lead to inaccuracy. So, we need to relax the assumption by moving from the diagonal matrix to the full-covariance matrix in our simulation.

Assuming $F = 4$ features and $N=50000$ samples in the simulation, the diagonal covariance matrix has *F* variance and each component has *F* mean parameters and 1 mixing weight; the parameters used will be $p = K\times F + K\times F + K-1$. So, the parameters needed for $K=3$ is 26. On the other hand, for the full covariance matrix, it becomes $K\times F + K\times \frac{(F)(F+1)}{2} + K-1$, and this value for $K=3$ will come to 44.

The ablation study that compares the Akaike information criterion (AIC) and Bayesian information criterion (BIC) for different *K* values and different covariance matrix structure is shown in Table 2. In the Table 2, AIC is caluclauted using $-2 \times \log (\text {Likelihood}) + 2p$ and BIC is calculated using $-2\times \log (\text {Likelihood}) + p \times \ln (N)$ respectively and the $\log \text {Likelihood}$ is computed using the syntax $\text {model.score(data)} \times N$.

**Table 2:** GMM model selection results: model comparison by AIC and BIC (N=50,000, F=4).

K	Covariance ($\Sigma$)	Log-Likelihood	p (Params)	AIC	BIC
2	diag	-21748.26	21	43538.51	43675.37
2	full	-15412.59	41	30907.19	31174.39
3	diag	-19561.06	26	39174.12	39455.51
**3**	**full**	**-13527.28**	**44**	**27142.56**	**27626.87**

The $K=3$ full covariance model achieves the lowest AIC and BIC indicating that this model best fits the dataset. So, it validates that feature independency is inappropriate. Hence, it is needed to simulate a full covariance structure-based dataset.

The models that we consider for comparison are namely, Non-ML reference model, logistic regression, random forest, gradient-boosted trees, and multilayer perceptron. The GMM is set with components $K = 3$ to represent different network conditions. The parameters used in the other models are shown in Table 3.

**Table 3:** Simulation parameters used in various models.

Method	Parameter	Value
Logistic regression	Maximum iteration	1000
Random Forest	No. of estimators	100
Gradient-boosted trees	No. of estimators	100
Multi layer perceptron	Hidden layer sizes	(10,5)
	Maximum iteration	500

The performance of each model is validated using the metrics, namely, ROC-AUC, PR-AUC, F1 score, accuracy, brier score and expected calibration error (ECE). These metrics for different models are compared with GMM, and the results are shown in Table 4.

**Fig. 3:**
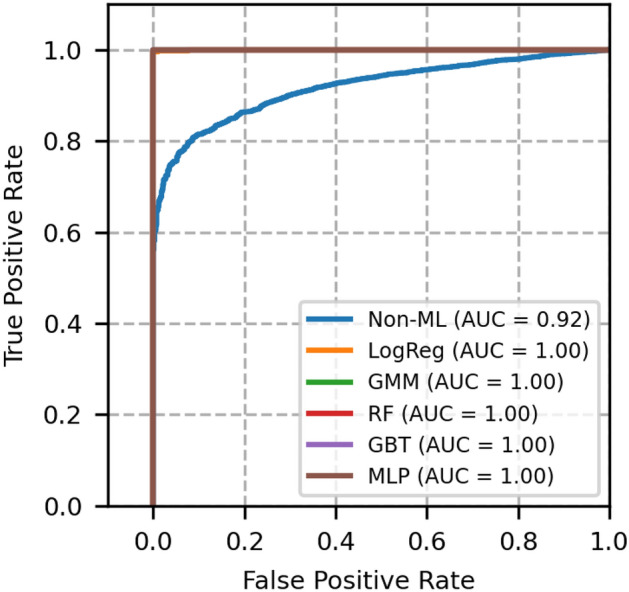
ROC curves of network topology 1- A comparison between GMM and other models.

**Fig. 4:**
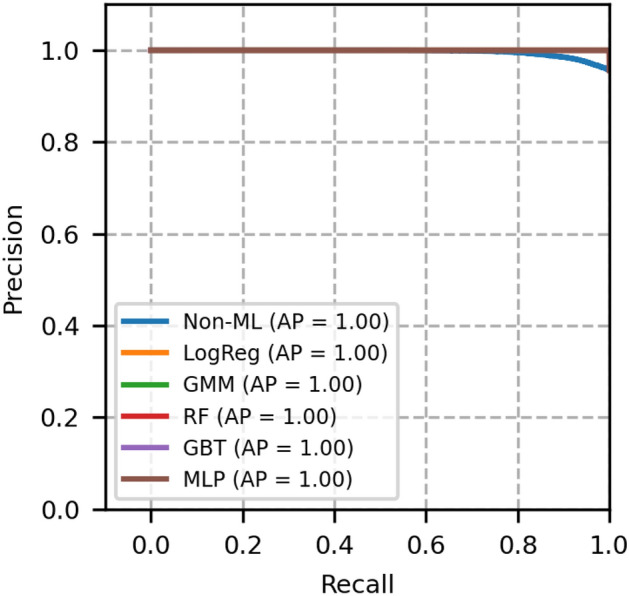
Precision recall curves of network topology 1- A comparison between GMM and other models.

**Fig. 5:**
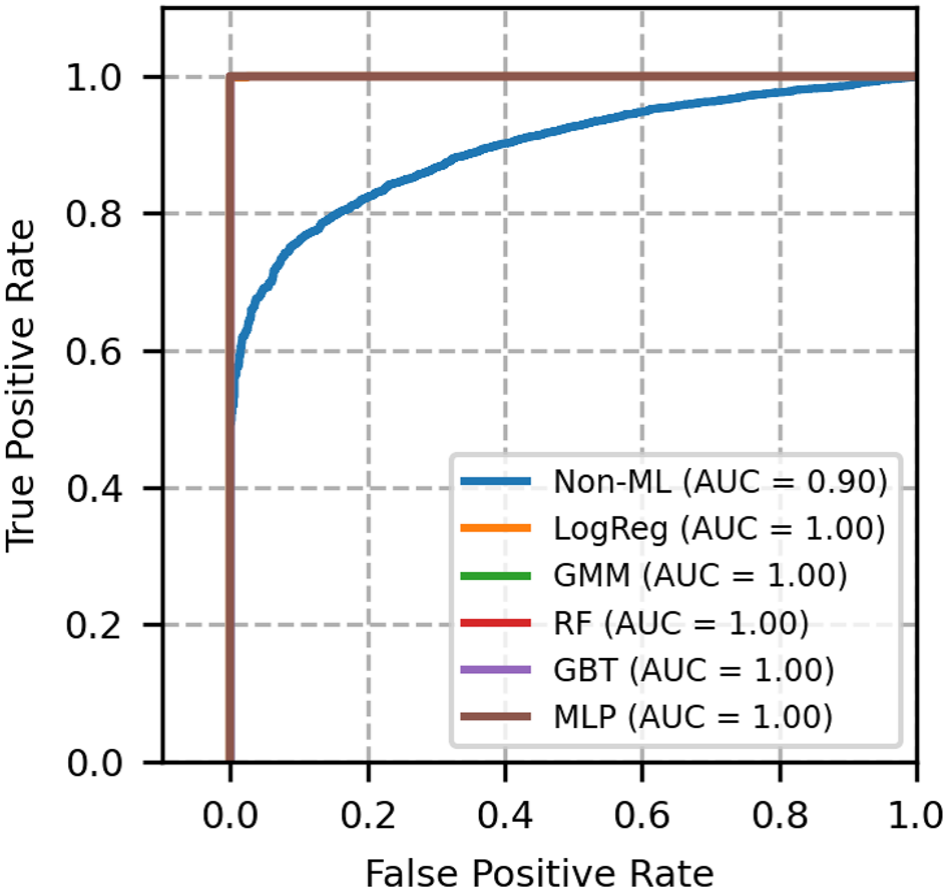
ROC curves of network topology 2- A comparison between GMM and other models.

**Fig. 6:**
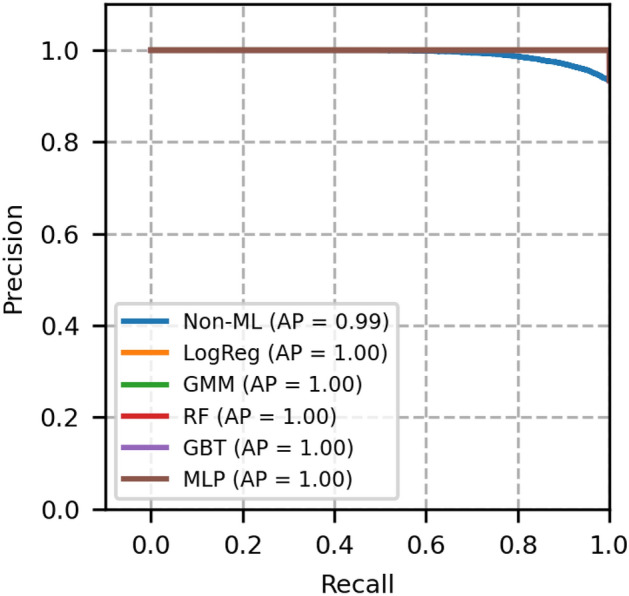
Precision recall curves of network topology 2- A comparison between GMM and other models.

**Fig. 7:**
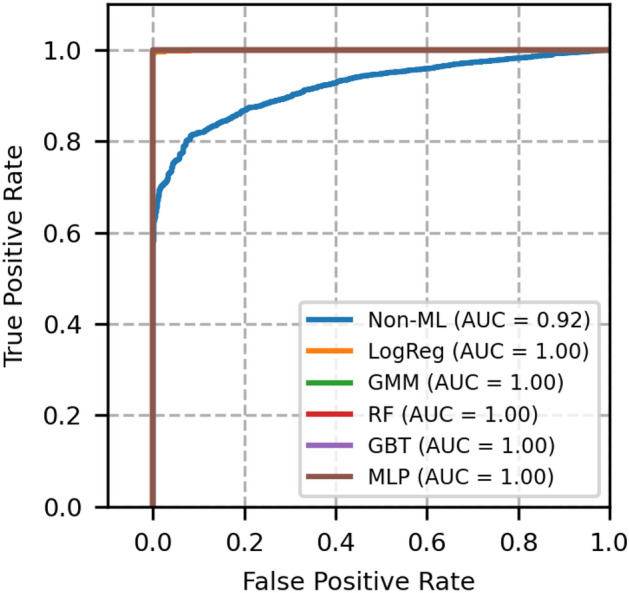
ROC curves of stress test of network topology 1- A comparison between GMM and other models.

**Fig. 8:**
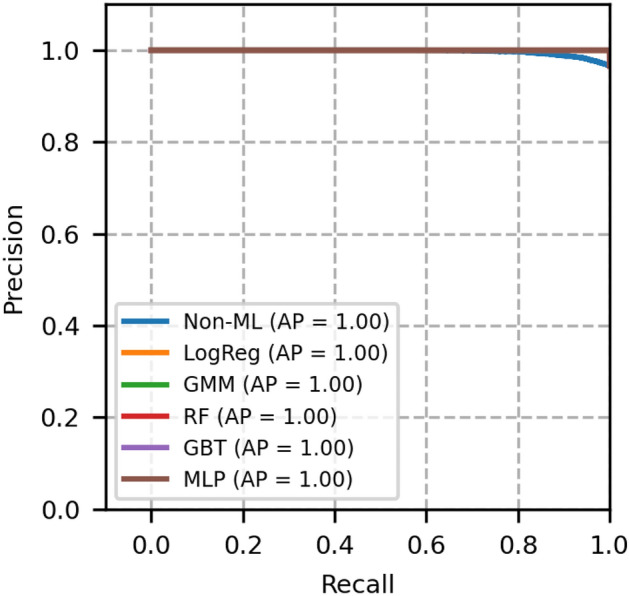
Precision recall curves of stress test- A comparison between GMM and other models.

**Fig. 9:**
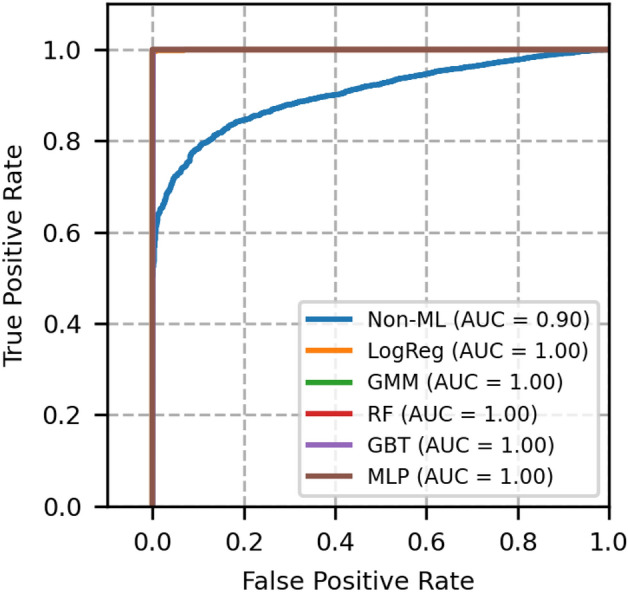
ROC curves of stress test of network topology 2- A comparison between GMM and other models.

**Fig. 10:**
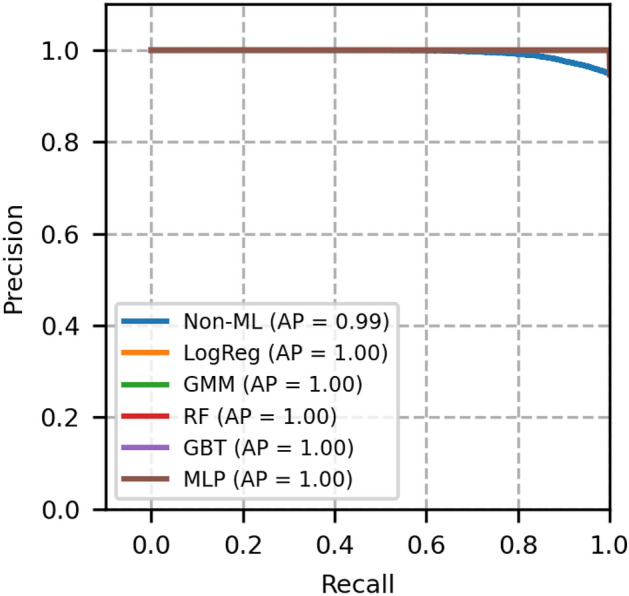
Precision recall curves of stress test of network topology 2- A comparison between GMM and other models.

**Table 4:** Comparative Performance of Models.

Model	ROC-AUC	PR-AUC	F1 Score	Accuracy	Brier Score	ECE
Non-ML	0.9153	0.9959	0.9778	0.9566	0.0407	0.0383
LogReg	0.9998	1.0000	0.9966	0.9936	0.0056	0.0142
GMM	1.0000	1.0000	0.9998	0.9995	0.0004	0.0005
RF	1.0000	1.0000	1.0000	1.0000	0.0000	0.0001
GBT	1.0000	1.0000	1.0000	1.0000	0.0000	0.0000
MLP	1.0000	1.0000	0.9992	0.9984	0.0012	0.0031

The plot of receiver operating characteristics (ROC) and precision recall (PR) of network topology 1 is shown in Figs. 3 and 4, in which all the machine learning-based models equally perform well. The area under the curve of these models is 1. A heuristic, formula based Non-ML model reflects an area under the curve value as 0.96, which serves to be a reference for comparison of other machine learning based models.

Moreover, from Table 4, we see that the Brier score and ECE score of GMM are less, i.e., 0.0004 and 0.0005 respectively, which is less than a few other models and is desirable. From these scores and from Figs. 3 and 4, it is obvious that GMM outperforms other methods collectively in ROC, PR, BER and ECE.

The ROC and PR plots of network topology 2 are shown in Figs. 5 and 6 respectively. This is a 15 nodes and 23 bidirectional links topology, for which *N* is chosen to be 50000 and the simulation is run for 100 random seeds. From this figure, it is known that the non-ML reference model offers slightly less ROC-AUC than that of topology 1. In addition, from these figures, we again confirm that the GMM outperforms non-ML based method and performs better or equally well with other ML based models.

### Robustness

#### Stress test

As the dataset are synthetic, we need to verify the robustness of our simulation results, by incorporating the changes in physical parameters. This is achieved by incorporating the following changes. While generating the SNR in the data generation step, the noise source is multiplied by a multiplier varying from 0.8 to 1.2 in a uniform distributed fashion. This ensures robustness against noise and device aging. In addition, the member of the ‘edges’ list in the python code is slightly perturbed before calculating the BER. This can ensure minimum errors against path length. Moreover, a non-linear term is introduced in the BER calculation that scales with the square of the transmission speed and distance. This enables the model to capture subtle physical effects beyond simple BER heuristics.

The results of the stress test in terms of ROC and PR is shown in Figs. 7 and 8 for topology 1 and in Figs. 9 and 10 for topology 2 respectively. There is not much compromise in the performance, while the datasets undergo the stringent requirements and changes as mentioned in the previous paargraph.

#### Cross topology transfer

**Fig. 11:**
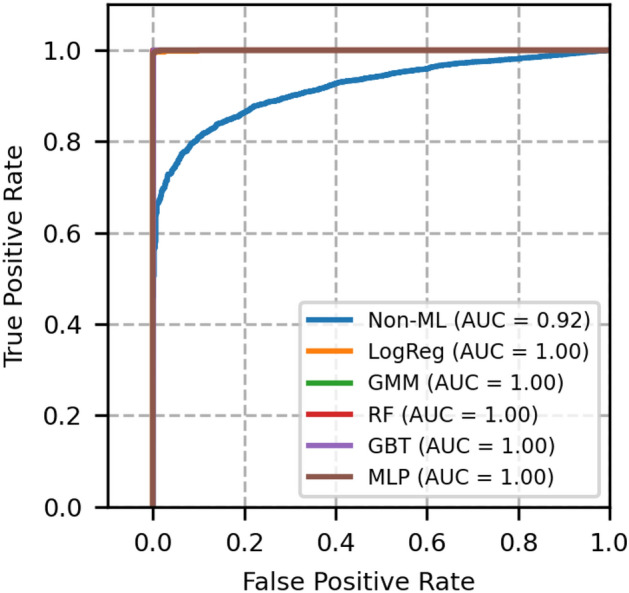
ROC curves of cross topology transfer - A comparison between GMM and other models.

**Fig. 12:**
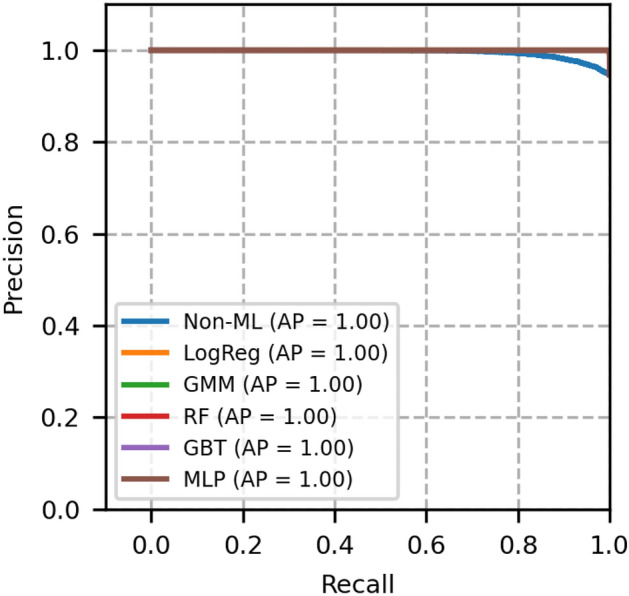
Precision recall curves of cross topology transfer- A comparison between GMM and other models during.

The cross topology transfer is another way of ensuring the robustness of GMM, when synthetic dataset is used in the simulation. To perform this test, the model is trained using the dataset generated from network of topology 1, while tested with the dataset generated from network of topology 2. Number of samples used for training are 50000 and number of samples used for testing purpose are 15000. Training samples are collected form topology 1 and testing samples are collected from topology 2 respectively. The ROC and precision-recall curves are plotted and shown in Figs. 11 and 12. While comparing with earlier performances shown in Figs. 3, 4, 5 and 6, we see no deviation in the performance. As the train and test datasets were used interchangeably for testing and training respectively, the slight deviation is obvious in non-ML reference model, but it does not affect the performance of GMM.

### Operational decision rule

The model’s output $P(Y = 1|F)$ is translated into an action, which could be accept or deny a light path. This decision making relies on the threshold $\tau$, which balances the tradeoff between false negative and false positives. If $P(Y=1|F) < \tau$, then the probability of failure is low, so that the deployment can be made. On the other hand, if $P(Y=1|F) \ge \tau$, then the route is blocked or rerouted. Instead of choosing the threshold as default value, like 0.5, it is obtained from the precision recall curve.

In a binary classification, a model’s uncertainty is measured using predictive entropy, which is defined as

8 $$\begin{aligned} E = P(Y=1|F) \ln (P(Y=1|F)) + P(Y=0|F) \ln (P(Y=0|F)) \end{aligned}$$

An entropy value, say $\ln (2) = 0.693$ ensures that the model is 50 % on its prediction, and therefore in high uncertainty.

The decision region can be expanded into three zones, using $\tau _h$, an upper and $\tau _l$, a lower threshold as shown in (9),

9 $$\begin{aligned} \text {Decision} = {\left\{ \begin{array}{ll} \text {Accept/deny} & \text {if } E < \tau _{\text {l}} \\ \text {Trigger Optical Performance Monitoring Measurement} & \text {if } \tau _{\text {l}} \le E \le \tau _{\text {h}} \\ \text {Deny Lightpath} & \text {if } E > \tau _{\text {h}} \end{array}\right. } \end{aligned}$$

### Computational footprint and latency

Integration of any model requires low latency for real time decision making. The latency of the model will be of the order of few ms. Moreover, the model GMM, after training will require a small storage space in terms of a few KB to occupy a RAM or disk. So, it offers extremely fast predication. Also, the model can be exported as a ONNX or Pickle files and deployed within a fast language environment, like C++ or Java.

## Limitation and future work

The results presented rely on synthetic data generated from a simplified physical model, which inherently introduces a domain gap when translating performance to a real-world deployed system. Future work will focus on integrating the techniques such as, adversarial learning or transfer learning—to fine-tune the trained models on small sets of real-world operational data. This approach can bridge the gap between simulated performance and robust deployment of these predictive models in production optical networks.

## Conclusion

This paper proposes a new, data-driven approach for the estimation of Bit Error Rate (BER) and Signal-to-Noise Ratio (SNR) in optical communication networks, grounded in deep learning and probabilistic modeling principles. With the use of Gaussian Mixture Models (GMM), the new approach precisely outlines the complex, nonlinear dependence of the main network parameters traffic volume, modulation format, transmission speed, and link length on the corresponding transmission quality parameters, i.e., BER and SNR. The use of GMM, trained and tested on typical datasets of Korean network topology, facilitates reliable estimation and classification of transmission quality over a wide range of network situations. Python based simulation results exhibit an impressive Area Under the Curve (AUC) value of 1.00, while maintaining the high accuracy, F1 score, low Brier score and low ECE. indicating the accuracy and reliability of the approach. The new approach not only enhances the prediction of transmission performance over undefined paths but also facilitates optimization of network planning, monitoring, and optimization in future-generation optical networks.

## Data Availability

The datasets used and/or analysed during the current study available from the corresponding author on reasonable request.

## Code availability

The code is available in the link https://github.com/Johnchristopherclement/GMM_Based_QoT_Enhancement.
